# *MEROPS*: the database of proteolytic enzymes, their substrates and inhibitors

**DOI:** 10.1093/nar/gkt953

**Published:** 2013-10-23

**Authors:** Neil D. Rawlings, Matthew Waller, Alan J. Barrett, Alex Bateman

**Affiliations:** ^1^The Wellcome Trust Sanger Institute, Wellcome Trust Genome Campus, Hinxton, Cambridgeshire CB10 1SA, UK and ^2^Proteins and Protein Families, EMBO European Bioinformatics Institute, Wellcome Trust Genome Campus, Hinxton, Cambridgeshire CB10 1SD, UK

## Abstract

Peptidases, their substrates and inhibitors are of great relevance to biology, medicine and biotechnology. The *MEROPS* database (http://merops.sanger.ac.uk) aims to fulfill the need for an integrated source of information about these. The database has hierarchical classifications in which homologous sets of peptidases and protein inhibitors are grouped into protein species, which are grouped into families, which are in turn grouped into clans. Recent developments include the following. A community annotation project has been instigated in which acknowledged experts are invited to contribute summaries for peptidases. Software has been written to provide an Internet-based data entry form. Contributors are acknowledged on the relevant web page. A new display showing the intron/exon structures of eukaryote peptidase genes and the phasing of the junctions has been implemented. It is now possible to filter the list of peptidases from a completely sequenced bacterial genome for a particular strain of the organism. The MEROPS filing pipeline has been altered to circumvent the restrictions imposed on non-interactive blastp searches, and a HMMER search using specially generated alignments to maximize the distribution of organisms returned in the search results has been added.

## INTRODUCTION

The *MEROPS* database is a manually curated information resource for proteolytic enzymes [For simplicity, we here use the term ‘peptidase’ for any proteolytic enzyme, although a few of them are not peptidases in the strictest sense because they are lyases and not hydrolases (1)], their inhibitors and substrates. The database can be found at http://merops.sanger.ac.uk. The organizational principle of the database is a hierarchical classification in which homologous sets of peptidase and protein inhibitor sequences are grouped into peptidase and inhibitor species, which are in turn grouped into families, which are grouped into clans. A family contains related sequences, and a clan contains related structures. Sequence analysis is restricted to that portion of the protein directly responsible for peptidase or inhibitor activity, which is termed the ‘peptidase unit’ or the ‘inhibitor unit’, respectively. A peptidase or inhibitor unit normally corresponds to a structural domain, and some proteins contain more than one peptidase or inhibitor domain. Examples are potato virus Y polyprotein, which contains three peptidase units, each in a different family, and turkey ovomucoid, which contains three inhibitor units all in the same family. At every level in the database a well-characterized type example is chosen, to which all other members of the family or clan must be shown to be related in a statistically significant manner. The type example at the peptidase or inhibitor level is termed the ‘holotype’ (2,3). There are usually three releases of the *MEROPS* database per year.

The sequence of family names is not consecutive because some families have been removed from the database. The most frequent reason why a family is removed is because a sequence relationship has been discovered to another family in the database. When the families are merged, the family name with the lowest number is retained and the one with the highest number is marked as deleted. A family may also be removed if experimentation has shown that the activity is not that of a peptidase. When a family is removed, the family name is not reassigned. A bookmarked link to a deleted family will either be automatically redirected to the new family name (or MEROPS identifier) or a message will appear to state that the family is no longer included in the database.

Statistics from release 9.9 (August 2013) of *MEROPS* are shown in Table 1 and compared with release 9.5 from July 2011. Counts of substrate cleavages, peptidase-inhibitor interactions and references are shown in Table 2.

**Table 1.** Counts of protein species, families and clans for proteolytic enzymes and protein inhibitors in the *MEROPS* database

	MEROPS 9.5		MEROPS 9.9	
	Peptidases	Inhibitors	Peptidases	Inhibitors
Sequences	192 053	17 451	413 834	28 502
Identifiers				
Experimentally characterized and sequenced	2308	518	2438	542
Hypothetical from model organisms	1250	0	1362	0
Not active as peptidase or inhibitor	298	117	327	115
Experimentally characterized but unsequenced	145	0	148	0
Pseudogenes	70	0	70	0
Compound and complex proteins	15	52	16	49
Total	4086	687	4361	706
Families	225	71	244	76
Clans	44	34	55	39

The numbers in Release 9.9 of *MEROPS* (August 2013) are compared with those in Release 9.5 of *MEROPS* (July 2011). A peptidase is referred to as ‘unsequenced’ when no sequence is known, or the known sequence fragments are insufficient to be able to assign the peptidase to a family

**Table 2.** Information in the MEROPS database

	MEROPS 9.5	MEROPS 9.9
Substrate cleavages: total	54 838	64 022
Substrate cleavages: physiological	18 280	20 591
Substrate cleavages: non-physiological	28 376	35 897
Substrate cleavages: pathological	990	1166
Substrate cleavages: synthetic substrates	4229	4906
Peptidase-inhibitor interactions: total	4017	4485
Peptidase-inhibitor interactions: proteins	1220	1304
Peptidase-inhibitor interactions: SMI	2373	2562
References	43 497	52 600

Substrate cleavage totals do not include cleavages derived only from the SwissProt database (mainly removal of initiating methionines and signal peptides). A naturally occurring cleavage is described as ‘physiological’ when the peptidase and substrate are from the same organism and ‘pathological’ if the organisms differ and are pathogen and host. More than half of the cleavage positions in the MEROPS collection have been identified by mass spectroscopy, of which over 4800 cleavages were obtained from the PRIDE database (4) and over 3100 from the TOPPR database (5). Over 3300 cleavages were derived from the CutDB database (6). Molecular Connections (Bangalore, India) have provided over 10 000 cleavages collected from the literature. How these data have been annotated has been described previously (7)

### Finding homologues

To find homologues for a family we have performed blastp searches (8), usually using the non-interactive facilities at the National Center for Biotechnology Information (NCBI), searching the non-redundant protein sequence database (9). However, a number of families have now exceeded 10 000 homologues, which is the maximum number returned from a blastp search at NCBI. These include the families C26 (the family of gamma-glutamyl hydrolase), C44 (amidophosphoribosyltransferase precursor), M16 (pitrilysin), M20 (glutamate carboxypeptidase), M23 (beta-lytic metallopeptidase), M24 (methionyl aminopeptidase), S1 (chymotrypsin), S9 (prolyl oligopeptidase) and S33 (prolyl aminopeptidase). Some of these families have exceeded 20 000 homologues (C26, S1 and S9), and family S12 (d-Ala-d-Ala carboxypeptidase B) is approaching 10 000 homologues. The reasons why a family contains so many homologues vary, for example, methionyl aminopeptidase removes the initiating methionine from cytoplasmic proteins and is present in every genome so far sequenced; there have been numerous gene duplications in vertebrates and insects for family S1 (the human genome contains 186 homologues, and *Drosophila melanogaster* 307 homologues). Some families contain relatively few peptidases and many homologues that are termed ‘non-peptidase homologues’; for example, family S9 contains 5780 homologues that are not peptidases, usually because one of the active site residues has been replaced, but are other kinds of enzyme that have the ‘α/β hydrolase’ fold, such as lipases, carboxylesterases and esterases.

To keep the peptidase and peptidase inhibitor families up-to-date with current genome sequencing projects, an addition to blastp searches was sought. For release 9.9, a second search was performed: the sequence filing pipeline (10,11) was modified so that the initial blastp search was replaced by a search of the NCBI non-redundant protein sequence database using HMMER as implemented at Janelia Farm, Howard Hughes Medical Institute (http://hmmer.janelia.org/) (12). HMMER searches allow submission of a sequence alignment, and for this purpose special alignments were generated for each family and subfamily in MEROPS.

Because we wished to find homologues from the widest range of organisms possible, we generated a special alignment by selecting an example from every phylum that is represented in a peptidase family or subfamily. Where possible, sequences from different MEROPS identifiers, thus representing different peptidase species (11), were used. For example, the alignment for subfamily A1A contained homologues from 12 different phyla (see Table 3). So that the HMMER search can be repeated by others, the sequences used for each family or subfamily are flagged in the MySQL database, which can be downloaded from our FTP site. Each alignment was generated using ClustalX (13).

**Table 3.** Example of sequences used in an alignment submitted to the HMMER server

Organism	Phylum	MEROPS identifier	Accession	Residue range
Human	Chordata	A01.070	B4DVY9	63–388
*Drosophila melanogaster*	Arthropoda	A01.A66	Q9VEK4	51–370
*Saccoglossus kowalevskii*	Hemichordata	A01.009	XP_002731917	55–386
*Strongylocentrotus purpuratus*	Echinodermata	A01.096	XP_780533	66–310
*Capitella capitata*	Annelida	A01.009		12–343
*Caenorhabditis elegans*	Nematoda	A01.A73	CAB60913	56–320
*Schistosoma mansoni*	Platyhelminthes		G4VG04	58–336
*Hydra magnipapillata*	Cnidaria	A01.006	XP_002154870	92–417
*Trichoplax adhaerens*	Placozoa		B3RK54	16–344
*Amphimedon queenslandica*	Porifera		XP_003385244	56–379
*Arabidopsis thaliana*	Streptophyta	A01.A33	O65453	33–335
*Meloidogyne incognita*	Rhodophyta	A01.053		82–406
*Chlamydomonas reinhardtii*	Chlorophyta	A01.096	Q7XB41	65–307, 490–578
*Phaeodactylum tricornutum*	Ochrophyta		B7FZ37	86–448
*Ectocarpus siliculosus*	Heterokontophyta		D7FLX5	93–407
*Phytophthora infestans*	Oomycota		D0N6R0	25–378
*Coprinus cinereus*	Basidiomycota		A8N6S9	143–366
*Saccharomyces cerevisiae*	Ascomycota	A01.018	P07267	78–405
*Rhizopus oryzae*	Zygomycota		I1BX70	57–254
*Batrachochytrium dendrobatidis*	Chytridiomycota	A01.018	F4NZG7	69–399
*Dictyostelium discoideum*	Sarcomastigophora	A01.A89	O76856	50–378
*Trichomonas vaginalis*	Parabasalidea		A2FIM5	44–351

The identifiers for the sequences used to generate an alignment for family A1 subfamily A are shown. Where no MEROPS identifier is listed, it is because a putative peptidase was used that could not be mapped to a MEROPS identifier. Accessions cited are mainly UniProt or RefSeq or are Protein Identifiers. The sequences from *Capitella capitata* and *Meloidogyne incognita* are translations from the genes *Capca1_225009* and *Minc12021*, respectively. The residue range of the peptidase domain is given; in the case of Q7XB41, an unrelated nested domain interrupts the peptidase domain.

The results from the HMMER searches returned more hits, but otherwise were consistent with the blastp searches in that all the hits found by blastp were also found by HMMER. The MEROPS filing pipeline was otherwise unchanged. Each sequence was submitted to a local blastp search against the MEROPS sequence collection, so that the extent of the peptidase domain and active site residues could be calculated and a MEROPS identifier could be assigned.

If a peptidase or protein inhibitor family contained homologues from only one phylum, or contained only sequences from viruses, then only a blastp search was performed.

The methods for collecting homologues will change in the future because there is still a limit (20 000 sequences) on the number of homologues returned by the HMMER search implemented on the HMMER web server.

As can be seen from Table 1, the number of sequences in MEROPS has more than doubled since July 2011. We reported a similar doubling in sequences between April 2007 and August 2009 (14), but a more moderate increase between August 2009 and July 2011 (15). The most recent doubling of sequences is partly due to the ability of HMMER searches to find additional distantly related homologues and also the increase in the number of completely sequenced genomes.

### MEROPS community input

Table 1 shows that the number of peptidases that can be distinguished now exceeds 4000, each of which has been assigned a unique MEROPS identifier. Some of these identifiers have been set up for particular model organisms that have been the subject of genome sequencing projects, and the peptidase homologues have not yet been biochemically characterized (16). If these putative proteins are excluded, then the number of distinct biochemically characterized peptidases in release 9.9 is 2646. There is a computer-generated summary for each of these, showing the MEROPS classification, a figure showing the domain architecture and, if enough substrate cleavages are known, displays of specificity. In addition, there are pages for all orthologous proteins showing a dynamically generated alignment, a list of primary database cross-references (protein and nucleotide), a list of active site residues, a display of distribution amongst organisms, cross-references to entries in the Protein Data Bank (17,18) and a Richardson diagram (19) if a tertiary structure has been solved, a bibliography, a list of substrates and their cleavage sites, a list of interactions with protein and small molecule inhibitors and cross-references to databases of pharmaceutical interest. There is, however, very little text.

MEROPS is run by a small team, and it is not possible for members of the team to write and maintain over 2600 peptidase summaries. This is an ideal project for the wider scientific community. Community annotation projects have either made use of a centralized database such as Wikipedia, which is freely open to the general public or have used a system of registration so that only experts can contribute and the contribution is acknowledged. A successful example of a project using Wikipedia has involved the Rfam database of non-coding RNA sequences (20). A successful community annotation project that invites experts to contribute has been Reactome, which features biological pathways that include enzymes (21). We have chosen to follow the latter model.

The MEROPS community annotation project requires a consultant to register to receive a unique password. To log in, a consultant must provide an email address and password. The consultant is then presented with a list of MEROPS identifiers and their recommended names, which are the pages available to edit. Should a consultant wish to add a peptidase to his or her list, then he or she can request this.

On clicking the ‘edit’ button, the consultant is presented with a (usually) blank form with the following headings: name and history, pH optimum, activity and specificity, RNA splicing, preparation, physiology, pharmaceutical relevance, biotechnology, biological aspects, subcellular location, knockout, distinguishing features, substrates (which links to the list of known cleavages in substrates), inhibitors (which links to the list of known peptidase/inhibitor interactions), special substrate and special inhibitor. All of these sections are available for editing, but some may contain text added by the MEROPS curators (especially the physiology, pharmaceutical relevance, biotechnology and knockout fields). A consultant is not expected to enter text for every field, and if no information is known the field is best left empty.

When a consultant has completed his or her edits and wishes the summary to appear in the next release of MEROPS, then he or she can select ‘Review Requested’ in the ‘Review stage’ menu and then save the page. The MEROPS identifier is added to the list of pages submitted for review, which is only visible to the curators.

**Figure 1.**
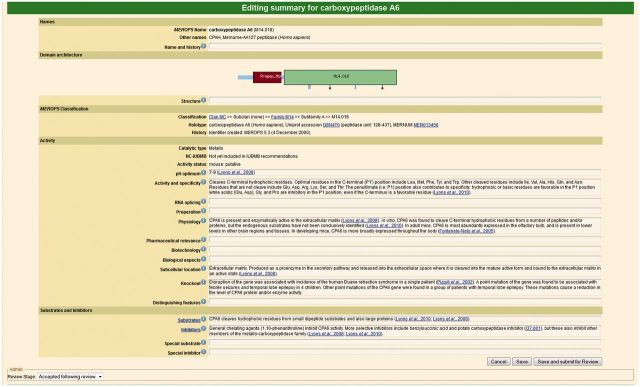
Form for the submission of a peptidase summary for the MEROPS community annotation project. The summary for carboxypeptidase A6 (MEROPS identifier M14.018) is shown. The summary was kindly provided by Professor Lloyd Fricker.

The MySQL database stores all saved versions of each section of each summary. The final summary presented to the administrator will be the most recently saved version of each section. Once reviewed by the administrator, the summary can be imported into the main MEROPS MySQL database. The curator adds the author details (names and affiliations) and the finished summary will appear in the next release of the MEROPS database. The administrator then resets the review stage to ‘Incomplete’ and the summary is again available for the consultant to edit. An example of a completed summary is shown in Figure 1.

Following the publication of the third edition of the Handbook of Proteolytic Enzymes (22), which contains chapters on over 800 peptidases, each written by one or more acknowledged experts, the authors of each chapter were invited to contribute to the MEROPS community input project in March 2013. To date, we have received over thirty summaries that have now been included on the MEROPS website.

### Recent developments

*Gene displays*. Comparisons of the intron–exon structure of eukaryote genes have proved to be useful in understanding their evolution. It had been noticed that within vertebrates, gene duplications frequently occurred after the insertion of introns, so that the exon/intron structure is preserved amongst paralogues. A theory for how regions of DNA coding for specific domains could be shuffled between one gene and another was developed by Patthy (23). A new display to present gene structures has been added at the peptidase level. The display shows the known exon and intron structure for a eukaryote gene. An exon is shown as a box and is numbered. Introns are shown as the thick line between the exons. The phase of the intron is indicated above the intron, where phase 0 means the intron is inserted between codons, phase 1 between the first and second base of the triplet and phase 2 between the second and third base of the triplet. All gene structures are taken from research articles where the structure was experimentally determined and are not taken from genome sequencing projects, where there may be problems with misidentification of exon–intron junctions, omission of exons and erroneous insertion of introns into coding sequence. The gene sequence displayed is from the initiation ATG to the stop codon, so introns within 5′ and 3′ untranslated regions are not shown. Alternatively spliced variants are shown where they have been experimentally proved to exist. Peptidase and protein inhibitor gene structures have been collected from the following eight model organisms: human, mouse, rat, *Drosophila melanogaster*, *Caenorhabditis elegans*, *Arabidopsis thaliana*, *Saccharomyces cerevisiae* and *Schizosaccharomyces pombe*. An example of the new display is shown in Figure 3.

**Figure 2.**
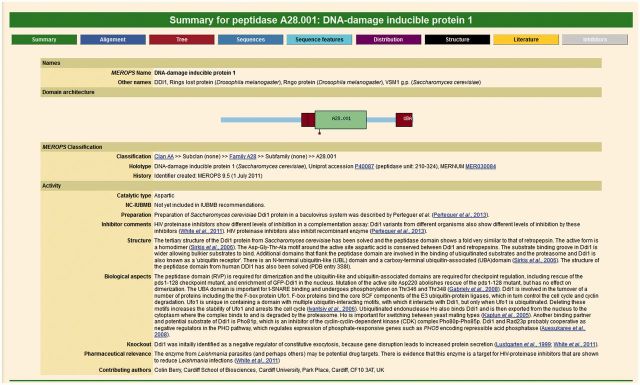
Example of a complete peptidase summary. The summary for DNA-damage inducible protein 1 (MEROPS identifier A28.001) is shown. The summary was kindly supplied by Dr Colin Berry.

*Organism pages*. It has become common practice to sequence the genomes of several different strains of the same bacterial species. The list of strains with completely sequenced genomes can now be displayed on the species page. Selecting one of the strains causes the results to be filtered, and only those peptidases or inhibitors present in that strain are displayed. It should be noted that the genome analysis at the foot of the page displays results for the selected strain and not the species.

*Peptidases from model organisms*. The number of model organisms has been increased to 11 with the addition of a Gram-positive bacterium (*Bacillus subtilis*), an archaean (*Pyrococcus furiosus*), a protozoan (*Dictyostelium discoideum*) and another yeast (*Schizosaccharomyces cerevisiae*). A special *MEROPS* identifier, in which the first character after the dot is A, B or C, has been created for each putative peptidase from each of these organisms.

*Literature*. Links are now being presented to Europe PubMed Central and PubMed.

A new item has been added to the search menu that allows a user to retrieve references by submitting a simple text search. A user can enter an author name, a term from a title or a journal name. The retrieved list displays the full reference with, where available, links to PubMed, PubMed central, the full text of the article and clan, family, peptidase or inhibitor summaries in *MEROPS*.

*Peptidase families and identifiers*. There have been two significant developments concerning peptidase family names and MEROPS identifiers.

The recent crystal structure of the precursor of the pantetheinyl hydrolase ThnT from *Streptomyces cattleya* (24) has shown that auto-activation exposes a threonine at the new N-terminus, occupying the same position as a serine in the homologous aminopeptidase DmpA from *Ochrobactrum anthropi*. This means that the nucleophile in peptidases in this family can be either threonine or serine. In all other known families of peptidases, the nucleophile is absolutely conserved. This means that the family cannot be named according to the convention used so far in *MEROPS* in which the first letter of the family name represents the nature of the nucleophile. This family has been named P1, which is the first in a new category of families with mixed nucleophiles.

**Figure 3.**
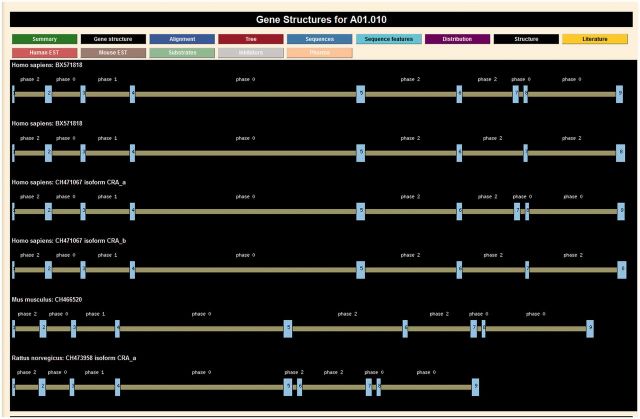
Example of a gene structure. The gene structures for cathepsin E (MEROPS identifier A01.010) are shown.

The first family to be assigned an identifier with three digits is the cysteine peptidase family C101, with includes the FAM105B (or OTULIN) isopeptidase (C101.001). This is a de-ubiquitinating enzyme that is specific for Met1 linkages (25).

## FUNDING

Wellcome Trust [WT0077044/Z/05/Z]. Funding for open access charge: Wellcome Trust.

*Conflict of interest statement*. None declared.
